# Characteristics of Spinal Cord Injury Presenting to Tertiary Care Center in Gandaki Province of Nepal: A Descriptive Cross-sectional Study

**DOI:** 10.31729/jnma.8619

**Published:** 2024-06-30

**Authors:** Yadu Nath Baral, Rajesh Kumar Yadav, Sushila Baral, Srijana Poudel, Yoveen Kumar Yadav, Subash Gurung, Kathit Raj Ghimire, Jhapindra Pokharel, Ashim Gurung

**Affiliations:** 1 Department of Orthopaedics and Trauma Surgery, Pokhara Academy of Medical Sciences, Western Regional Hospital, Gandaki province, Nepal; 2 Department of Global Fund, Save The Children International, Nepal; 3 Department of Public Health, School of Health and Allied Sciences, Pokhara University, Pokhara, Nepal; 4 Department of Medicine, Pokhara Academy of Medical Sciences, Western Regional Hospital, Gandaki province, Nepal; 5 Department of Public Health, Hope international college and Kantipur college, Kathmandu, Nepal; 6 Department of Neurosurgery, Pokhara Academy of Medical Sciences, Western Regional Hospital, Gandaki province, Nepal

**Keywords:** *epidemiology*, *trauma*, *spinal cord injury*

## Abstract

**Introduction:**

Spinal cord injury is one of the common injury which causes damage to the spinal cord due to trauma, diseases or degenerations leading to disability and decreasing life expectancy. The study aims to find the characteristics of spinal cord injury presenting at a tertiary care centre.

**Methods:**

A descriptive cross-sectional study was conducted at Pokhara, Gandaki Province, from 28^th^ March to 25^th^ September, 2023. 139 participants were interviewed for the study. Structured interview schedule and validated questionnaires were used for data collection. Ethical approval was taken for the study (Reference number: 151/079).

**Results:**

A total of 139 cases were observed; most common affected age group was between 25-55 years with a mean age of 48.68 years. Most 93 (66.91%) of the spinal cord injury patients were not enrolled in health insurance program. Most 107 (76.97%) common cause for spinal cord injury was falls from height. Age, gender, occupation and duration of stay in the hospital were statistically significant with mode of treatment.

**Conclusions:**

Spinal cord injury mostly traumatic commonly due to fall from height affecting mainly male population residing in rural areas at their fourth decade of life who are mainly involve in manual work and agriculture. Spinal cord injury is a major health problem at global and local level causing major morbidity.

## INTRODUCTION

Spinal cord injury is one of the common injury which cause damage to the spinal cord due to trauma, diseases or degenerations.^1^ Traumatic Spinal Cord Injury (TSCI) leads to social, economic burdens not only to the individuals but also to society and nation.^2^

Spinal cord injury lead to disability and decrease life expectancy.^3^ The management of spinal cord injury (SCI) requires significant health care resources and can place a substantial financial burden on patients, their families, and the community.^4^ Although injury prevention initiatives have attempted to reduce the occurrence of TSCI, the incidence and prevalence of TSCI have been increasing worldwide.^5^ The epidemiological characteristics of TSCI vary across countries, regions with different economic levels.^6-7^

Spinal cord injury (SCI) affects conduction of sensory and motor signals across the site of lesion, as well as the autonomic nervous system.^8^'^9^ The aim of this study was to determine the characteristics of spinal cord injured patients presenting to tertiary care center of Gandaki province.

## METHODS

This was a descriptive cross-sectional study which was done among the spinal cord injured patients. The study population were all the spinal cord injured patients who visited Western Regional Hospital, Pokhara Academy of Health Sciences of Gandaki Province of Nepal from 28^th^ March to 25^th^ September, 2023. Ethical approval was obtained from the Institutional Review Committee of Pokhara Academy of Health Sciences (Reference number: 151/079).

Spinal cord injury patient aged 18 years and above were considered as the study participants. Participants were fully informed regarding study objectives and written consent was obtained before the initiation of the data collection. Data was collected by face-to-face interview method from the Spinal cord injury patients using interview schedule in Nepali version at one point in time for each of the patients.

Patients response was closely recorded into the tool. Data was entered in Epi Data software and analysis was performed with the help of the Statistical Package for Social Science (SPSS). Univariate analysis was computed to describe socio-demographic profile of participants, level of injury, duration of injury, duration of hospital stays of spinal cord injury patients, while mean and standard deviation was calculated for continuous variables.

## RESULTS

Total of 908 patients presented to Western regional hospital due to traumatic injuries during study period, out of which 610 (67.18%) were male and 298 (32.82%) were female, 139 (15.30%) patients meet our inclusion criteria were further evaluated for study.

Among total spinal cord injury patient, male to female ratio was 3.2:1, 70 (50.36%) of the patient was in the age group 25-55 years (Table 1).

**Table 1:** Socio-demographic Characteristics of the Participants (n = 139)

Characteristics	n (%)
**Gender**	
Male	106 (76.26)
Female	33 (23.74)
**Age**	
<25 Years	17 (12.23)
25-55 Years	70 (50.36)
>55 Years	52 (37.41)
**Religions**	
Hindu	91 (65.47)
Buddhism	31 (22.30)
Christianity	17 (12.23)
**Permanent Residence**	
Urban	52 (37.41)
Rural	87 (62.59)
**Occupational**	
Labor	24 (17.27)
Agriculture	23 (16.55)
Unemployed	21 (15.11)
House Keeper	19 (13.67)
Business	17 (12.23)
Private Employee	11 (7.91)
Driver	2 (1.44)
Government job	6 (4.32)
Other(Students, Abroad)	16 (11.51)
**Health Insurance Status**	
No	93 (66.91)
Yes	46 (33.09)

Among 139 patients, 112 (80.58%) had fall injury, outof which 13 (9.35%) suatained injury dur to fall fromtree. Mean duration of injury in days before admissionin hospital was found to be1.92±1.92 days withmaximum delay in hospital admission found to be 14days (Table 2).

**Table 2:** Mode of Injury, duration of trauma to hospital admission and Duration of Hospital stay (n=139)

Characteristic	n (%)
Mode of Injury	112 (80.58)
Fall from Height	107 (76.98)
Fall from Tree	13 (9.35)
Road Traffic Accident (RTA)	13 (9.35)
Violence	4 (2.88)
Impact with Falling Wooden Log	2 (1.44)
**Duration of injury in days**	
≤2 Days	112 (80.58)
>2 Days	27 (19.42)
Mean 1.92 ±1.92, Min:1 & Max:14	
**Duration of stay in Hospital**	
≤11 Days	82 (58.99)
>11 Days	57 (41.01)
Mean 11.87 ±9.65, Min:1 & Max:47	

Lumbar region was involvedin 55 (39.57%) and thoracic region in 49 (35.25%) of cases. Neurological assessment done after the accuracy of recovery from spinal shock showed that about half 71 (51.08%) of the cases had ASIA 'D' Neurology. Chest injury was found 21 (15.11%) of patient with with spinal cord injury. Among 139 cases, 84 (60.43%) under-went operative intervention while 55 (39.57%) were managed non operatively (Table 3).

**Table 3:** Mode of treatment. (n=139)

	Mode of Treatment	
Characteristics	Operative n (%)	Non-Operative n (%)
**Age**		
<25 Years	14 (82.4)	3 (17.6)
25-55 Years	50 (71.4)	20 (28.6)
>55 Years	20 (38.5)	32 (61.5)
**Gender**		
Male	69 (65.1)	37 (34.9)
Female	15 (45.5)	18 (54.5)
**Occupation**		
Manual Worker	47 (52.8)	42 (47.2)
Business	15 (88.2)	2 (11.8)
Gov Job/Private Job	12 (70.6)	5 (29.4)
Unemployed	10 (62.5)	6 (37.5)
**Duration of Stay in Hospital**		
<11 Days	30 (36.6)	52 (63.4)
>11 Days	54 (94.7)	3 (5.3)
**Duration of Injury days**		
<2 Days	65 (58.0)	47 (42.0)
>2 Days	19 (70.4)	8 (29.6)

## DISCUSSION

The knowledge of the epidemiology of spinal cord injury in the country is important for planning, allocating resources for treatment and rehabilitation of patients, and developing strategies for prevention of spinal cord injury.^10^ Epidemiological profile and Management of spinal cord injury in developed countries differ significantly from that of developing countries like Nepal because of various causes like geographical challenges, occupation, lack of appropriate infrastructures, unavailability of trained health personnel, lack of appropriate mode of transportation and lack of early appropriate intervention.^11,12^

The male-to-female ratio varied from 1.6:1 to 15.5 :1 in different studies.^13-16^ We found male to be three times more prone to spinal cord injury when compared with females. This could be due to the fact that males are mainly involved in sternous physical activities and are sole earnings members of family while females are confined to household activities which make female less prone to injury due to fall from height.^17^

The mean age of patient with spinal cord injury in our study is 48.68 years which was similar to study done by Paudel et al.^12^ In study done in India, seventy one percent were in the age range of 20-49.^18^ The study done by Parajuli et al. in Nepal showed all age groups were almost equally affected by SCI with age >50 years being most commonly involved.^17^ Our study showed that people residing in rural areas sustained spinal cord injury more than people residing in urban areas which is similar to the study done in Bangladesh which showed that 69.2% of the spinal cord injury patients were from village areas.^19^ Because of difficult landscape and dependence in agriculture and farming in uneven lands make them vulnerable to fall , also climbing trees for gathering fodder to cattle increases the risk of fall from tree.

Manual labor and people involved in agriculture account for one third of spinal injury patient, nature of the work make this group more prone to fall injury, accidental injuries like impact of the falling heavy wooden log into the back. Similar result was seen in study done in India by N Mathur where 45 % of the spinal injury patient were involved in Farming (23.3%) and laborers (22.9%).^18^

Only one-third patient with spinal cord injury were enrolled in Health Insurance. Considering the cost of investigations, treatment and rehabilitation spinal cord injury is a costly morbidity and health insurance could be a means to make treatment accessible for poor and marginalized people. Furthermore Government of Nepal, in 2014, established an Impoverished Citizens Service Scheme of Social Health Security in which a spinal cord injured patient is covered with treatment expenses of a maximum of 1 lakh Nepalese rupee.^17,20^ This program could have been a boon to people of Gandaki province but such program has not been implemented in government hospital of Gandaki province, so people have been deprived of this government facility and have to struggle alone against the complications of spinal cord injury.

The most common cause of spinal cord injury in developed countries found to be road traffic accident while fall injuries found to be the most common cause of spinal cord injury in developing countries.^12,14,21^ In our study 77% patient sustained spinal cord injury due to fall from height while only 9.4% patient sustained spinal cord injury from road traffic accident which corresponds with findings of study done in Chitwan.^12^ Fall injuries were more common mode of spinal injury than road traffic accident in study done by N Mathur et al in India.^18^ Mostly the manual worker and those involved in farming and rearing cattle are the one to sustain fall injury leading to spinal cord injury.

The study done in national trauma centre found similar result which showed lumbar vertebra as commonly involved vertebra followed by dorsal vertebra.^11^ But the study done by N Mathur et al. found cervical vertebra to be most commonly affected followed by thoracic.^18^ Study done by Poudel et al showed the cervical region was the most commonly involved region followed by the lumbar.^12^ Fall injury result in more thoracolumbar injury while RTA cause more cervical injury.^22^

Our study found that 82.7% patient with spinal cord injury had incomplete neurology, and 17.3% had complete Neurological involvement. Similar result was found in the study done by Poudel et al. which showed 27 % had complete neurological involvement while 73 had incomplete neurology.^12^

Two-third of the patient sustained only spinal cord injury while one-third had other associated injuries of which chest was most commonly injured followed by head and limb injury respectively. The study done by N Mathur et al. showed the associated injury in only 5% of the cases and attributed lower cases of RTA as a cause of low number of associated injury.^18^ Chest injuries causes difficulty in chest physiotherapy and limb injuries require immobilization and some time stractions which increase the incidence of pressure sore and adversely affect the rehabilitation.^18^ Our study found that 60% of the patient underwent operative intervention while 40% underwent non operative treatment . Similar result found in the study done by Poudel et al.^12^

Our study has some limitations. It is a single tertiary centre descriptive cross-sectional study with short duration and limited number of sample.

## CONCLUSIONS

In present study majorly cause of spine injury was fall from height followed by fall from tree and road traffic accidents affecting mainly male population residing in rural areas at their fourth decade of life. The productive age group falls under high risk group. Hospital stay day is longer among the patients.
